# Inhibition of PTEN Ameliorates Secondary Hippocampal Injury and Cognitive Deficits after Intracerebral Hemorrhage: Involvement of AKT/FoxO3a/ATG-Mediated Autophagy

**DOI:** 10.1155/2021/5472605

**Published:** 2021-01-18

**Authors:** Manman Zhao, Junling Gao, Changmeng Cui, Yanan Zhang, Xiaohua Jiang, Jianzhong Cui

**Affiliations:** ^1^Department of Histology and Embryology, North China University of Science and Technology, Tangshan 063210, China; ^2^Department of Neurosurgery, Affiliated Hospital of Jining Medical University, Jining Medical University, Jining 272000, China; ^3^Department of Neurosurgery, Tangshan Gongren Hospital, Tangshan 063000, China

## Abstract

Spontaneous intracerebral hemorrhage (ICH) commonly causes secondary hippocampal damage and delayed cognitive impairments, but the mechanisms remain elusive. Here, we sought to identify the molecular mechanisms underlying these hemorrhagic outcomes in a rat autologous blood model of ICH. First, a significant increase in phosphatase and tensin homolog (PTEN) expression was observed in nonhemorrhagic ipsilateral hippocampus. However, systemic administration of PTEN inhibitor BPV or hippocampal injection of PTEN siRNA could prevent hippocampal neuronal injury and cognitive dysfunctions after ICH. Furthermore, we also found that ICH robustly triggered autophagic neuronal death in the ipsilateral hippocampus, but which were strongly reduced by PTEN knockdown. Notably, suppression of autophagy effectively attenuated poststroke hippocampal inflammation, neuronal damage, and cognitive decline, suggesting the beneficial effects of PTEN deletion was associated with autophagy inactivation. Specifically, PTEN antagonized the PI3K/AKT signaling and downstream effector FoxO3a phosphorylation and subsequently enhanced nuclear translocation of FoxO3a to drive proautophagy gene program, but these changes were diminished upon PTEN inhibition. More importantly, lentivirus-mediated FoxO3a overexpression apparently abrogated the antiauotphagy effect of PTEN deletion via enhancing autophagy-related gene (ATG) transcription. Collectively, these results suggest that knockdown of PTEN alleviated progressive hippocampal injury and cognitive deficits by suppression of autophagy induction involving the AKT/FoxO3a/ATG axis after ICH. Thus, this study provides a novel and promising therapeutic target for the treatment of hemorrhagic stroke.

## 1. Introduction

Spontaneous intracerebral hemorrhage (ICH), the most lethal subtype of stroke, accounts for 15-20% of all strokes. ICH results in 30-50% mortality, and only 10-20% of all survivors remain independent after 6 months [1]. More than 70% of survivors live with locomotor dysfunction, and up to 60% of these patients experience delayed cognitive decline [2, 3]. Hematomas, mainly occurring in the basal ganglia, cause proximal or distal white matter injury including axonal damage, demyelination, and synapse changes, ultimately leading to severe motor impairment and poor prognosis in patients [4]. Besides, posthemorrhagic cognitive decline is one of the main complications during the chronic phase of ICH. It is well-known that the hippocampus plays a critical role in the formation and maintenance of cognitive function. Although the hippocampus is not in situ hemorrhagic focus, a secondary injury may cause a decline of memory and cognition ability [5, 6]. More recently, a clinical study has shown that ICH patients present a progressive cognitive deficit as early as the 1st week, which is correlated with the fractional anisotropy values for the ipsilateral hippocampus [7]. Furthermore, numerous preclinical studies observed cognition, emotion, and depression-related behavioral deficits in rodent models of ICH [8–10]. These findings suggest that ICH causes functional dysfunction in distal brain regions, especially in the hippocampus. However, little is understood about the potential mechanisms responsible for secondary hippocampal injury and defective cognition after a hemorrhagic stroke.

Autophagy is a conserved catabolic pathway for the maintenance of cellular and metabolic homeostasis. As an intracellular self-degradation system, autophagy is generally considered an essential protective process that removes cytosolic components or damaged organelles [11]. Of note, autophagic cell death has been implicated in the pathogenesis of various diseases including neurodegenerative diseases and ischemic and hemorrhagic stroke [12, 13]. Recently, accumulating evidences have shown that iron released from the hematoma contributes to autophagy activation after ICH [14–16], but it is unknown that this process induces a beneficial or detrimental effect. Most studies support the notion that ICH-induced autophagy exacerbated histological and neurobehavioral consequences [13, 15, 17]. In contrast, Duan et al. found that activated autophagy exhibited neuroprotective effects against secondary brain injury via inhibiting endoplasmic reticulum stress at the early stage post-ICH [18]. Consequently, autophagy can display a prosurvival or prodeath role depending on the cellular context. Up to date, it is still unclear whether autophagy is triggered in distal brain regions after local ICH, particularly in the hippocampus.

Phosphatase and tensin homolog (PTEN), a tumor suppressor with dual-specificity phosphatase activities, plays a pivotal role in cellular survival, proliferation, energy metabolism, and genome maintenance [19]. Intriguingly, PTEN is known as a positive regulatory factor of autophagy by promoting autophagosome formation [20]. Also, inactivation of the PI3K/AKT/mTOR pathway has been implicated in PTEN-induced autophagy initiation [21, 22]. Previous study has indicated that PTEN oxidative inactivation strongly block phosphorylation of uncoordinated-51-like kinase 1 (ULK1) and ATG expressions to suppress myogenic autophagy [23]. Furthermore, Schwann cell-specific PTEN knockout mice display neuromuscular junction malformation, which is closely related to inhibition of mTOR-autophagy signaling [24]. An abundance of PTEN is required for neuronal differentiation, synaptogenesis, axonal branching, and regeneration in the central nervous system. Nevertheless, a significant increase of PTEN in the perihematomal tissues was observed in the early stage after ICH [25]. Inhibition of PTEN might obviously attenuate hemorrhagic brain injury in vivo and hemin-induced neuronal injury in vitro [26]. However, it has not yet been elucidated the regulatory effects and mechanisms of PTEN on autophagy stimulation in the hippocampus remote from the site of bleeding after ICH.

This study was conducted to determine whether the blockage of endogenous PTEN could alleviate secondary hippocampal injury and long-term cognitive deficits after basal ganglia hemorrhage, as well as the potential autophagic mechanism. This will provide insights into the biological and clinical significance of PTEN in hemorrhagic stroke.

## 2. Materials and Methods

### 2.1. Animals

All animal experiments were conducted in accordance with the Guide for the Care and Use of Laboratory Animals published by the National Institutes of Health (NIH). All protocols were approved by the Animal Care and Use Committee of Hebei Medical University. Adult male SD rats, weighing 300-320 g, were housed under conditions of constant temperature (23 ± 2°C) and humidity (55% ± 5%), in a 12 h light-dark cycle with free access to food and water. Enormous efforts were made to minimize animal suffering in the operation.

### 2.2. ICH Model

The ICH model was induced according to a previously described procedure [27]. Briefly, animals were anesthetized with 8% isoflurane in a 5 L/min oxygen flow in an anesthetic chamber and maintained with 4% isoflurane in a 2 L/min oxygen flow using a face mask. Rats were then fixed in a stereotactic instrument (Stoelting, USA) and infused 50 *μ*L autologous whole blood from the tail artery into the caudate nucleus of the right basal ganglia region of rats using a syringe pump (Harvard, USA) at a rate of 5 *μ*L/min. The coordinates were 0.2 mm anterior and 3.5 mm lateral to the bregma and a depth of 6.0 mm. The rats in the Sham group were injected with the same volume of saline.

### 2.3. Lentiviral Stereotaxic Injection

For specific knockdown on PTEN mRNA in the hippocampus, the lentiviral particles containing the siRNA targeting PTEN (LV-siPTEN) or scramble shRNA (LV-siControl) and recombinant lentivirus expressing FoxO3a (LV-FoxO3a) or its negative control oligonucleotide (LV-NC) were purchased from GeneChem Co. Ltd. (Shanghai, China). Rats received stereotactic microinjection of lentiviral particles at a dose of 2.0 *μ*L (10^8^-10^9^ TU/mL) into the CA1 region of the right hippocampus 72 h before ICH onset. The stereotaxic coordinates are as follows: anteroposterior −3.3 mm from the bregma, mediolateral ±1.6 mm from the midline, and dorsoventral −2.8 mm from the skull. The injection speed was 0.2 *μ*L/min, and the needle was left in place for 5 min after the injection. The transfection efficiency of the lentivirus was analyzed by real-time PCR and Western blot.

### 2.4. Drug Administration

ICH rats received an intraperitoneal injection of PTEN molecular inhibitor BPV (0.2 mg/kg, Sigma, MA, USA) or vehicle 6 h before ICH surgery, three times in total with an interval of 3 h. To assess the roles of autophagy on secondary histological and functional outcome, autophagy inhibitor chloroquine (CQ, 60 mg/kg, Sigma) or the corresponding volume of vehicle (25% dimethylsulfoxide in PBS) was intraperitoneally infused 6 h prior to ICH onset.

### 2.5. Y-Maze Test

The Y-maze test was performed to evaluate spatial working memory at 7 d after ICH in rats. The Y-maze device consisted of three identical arms separated by 120° angles. The rats were placed on the starting arm of the Y-maze and allowed to randomly explore for 10 min. The spontaneous alteration was calculated as follows: %spontaneous alternation = [(number of alternations)/(total entries − 2)] × 100.

### 2.6. Morris Water Maze Test

Spatial learning and memory ability was assessed using the Morris water maze (MWM). In the acquisition trial (days 8-12), the animals were randomly placed into a quadrant start points and allowed to find a hidden platform within 90 s. Four trails per day were conducted for consecutive 5 days with an interval of 20 min, and the swimming paths, latency, and velocity were recorded. For the probe trial (day 13), the platform was removed and the rats were allowed to swim freely for 60 s, and the number of target platform crossings and percentage of time spent in the target quadrant were recorded in this test.

### 2.7. Passive Avoidance Test

The passive avoidance test (PAT) is applied to assay learning and memory ability at 14 d post-ICH. The apparatus consisted of two compartments (light and dark) with a guillotine door. In the training trial, each animal was placed in the light compartment, and upon moving into the dark compartment, it immediately received an electric shock. The test trial was performed 24 hours later, during which each rat was reevaluated in a similar manner. The time taken (error latency) to reenter the dark compartment and the number of electrical shocks (error times) within 5 min were recorded.

### 2.8. Histology and Immunohistochemistry

Brain tissues were fixed in 4% paraformaldehyde (PFA) for 24 h, dehydrated with gradient alcohol, embedded in paraffin, cut into 5 *μ*m-thick coronal sections, and then stained with hematoxylin and eosin (H&E). In addition, immunohistochemical staining (IHC) was performed in accordance with the instructions of the SABC immunohistochemistry kit (Beyotime, Shanghai, China). The sections were incubated overnight at 4°C with rabbit anti-LC-3 polyclonal antibodies (Santa Cruz, CA, USA, 1 : 50) and subsequently with horseradish peroxidase- (HRP-) conjugated antirabbit IgG for 1 h. Finally, diaminobenzidine (DAB) was used to reveal the immunohistochemical reaction, and the sections were observed under an optical microscope (BX51, Olympus, Tokyo, Japan).

### 2.9. Double Immunofluorescence Staining

The brains were fixed in 4% PFA for 24 h, immersed in 30% sucrose, embedded in OCT, and then sectioned on a cryostat (15 *μ*m, Leica CM1950).The frozen sections were permeabilized in 0.2% Triton-100 for 20 min and subsequently treated with donkey serum for 1 h. The sections were then incubated with a mixture of rabbit anti-PTEN/LC3 polyclonal antibody and mouse anti-NeuN/GFAP (Santa Cruz, CA, USA, 1 : 50) overnight at 4°C followed by incubation with a mixture of fluorescein-conjugated antirabbit IgG and antimouse IgG (Cell Signaling Technology, MA, USA, 1 : 2000) for 1 h in the dark. The nuclei were labeled with DAPI. Finally, the sections were observed under a confocal laser scanning microscopy (TCS SP8 STED, Leica, Germany).

### 2.10. Transmission Electron Microscopy

The rats were anesthetized and perfused with 2% PFA and 2% glutaraldehyde in 0.1 mol/L Sorensen's buffer. The samples were then postfixed in 3% glutaraldehyde and 1% osmic acid for 2 h. After complete dehydration in graded ethyl alcohol, the samples were infiltrated with propylene oxide, embedded in epoxy resin, sectioned using a thin slicing machine, and stained with 2% uranyl acetate and Reynold's lead citrate. Images were captured using a transmission electron microscopy (Hitachi, Tokyo, Japan).

### 2.11. ELISA Analysis

To evaluate the poststroke inflammatory response in the ipsilateral hippocampus, protein supernatant from hippocampal tissues of rats in each group was used to quantify the contents of TNF-*α*, IL-1*β*, and IL-6 using the corresponding ELISA kit (Boster, Wuhan, China) according to the manufacturer's instructions.

### 2.12. Quantitative Real-Time PCR

Total RNA was extracted by TRIzol reagent (Invitrogen, CA, USA), and reverse transcription reactions were performed using the Superscript First-Strand cDNA synthesis system (Invitrogen, CA, USA). Real-time PCR was performed with Mastercycler-realplex Real-Time PCR System (Eppendorf, Germany) using a One-Step SYBR Prime Script TM RT-PCR Kit II (Takara, Tokyo, Japan) according to the manufacturer's instructions. The 2^-△△ct^ method was applied to calculate the levels of mRNA expression relative to that of GAPDH, and the values were presented by relative quantity.

### 2.13. Cytosol-Nucleus Fractionation

Harvested cells from the hippocampal tissue were suspended in cytoplasmic lysis reagent (Beyotime, Shanghai, China) for 20 min and centrifuged at 2,500g for 5 min at 4°C. The supernatant contained the cytosolic fraction. The nuclear pellet was suspended in nuclei lysis buffer for 10 min in ice, and the suspension was sonicated for 3 min followed by centrifugation at 12,000g for 15min at 4°C. The supernatant contained the soluble nuclear fraction.

### 2.14. Western Blot Analysis

Hippocampal proteins were extracted using RIPA lysis reagent containing proteinase and phosphatase inhibitors and quantified with the BCA kit (Solarbio, Beijing, China). 30 *μ*g of total protein was separated by 10% SDS-PAGE gel and transferred to 0.45 *μ*m PVDF membranes. After blocking with 5% fat-free dry milk for 1 h, the membranes were incubated overnight at 4°C with primary antibodies (Santa Cruz, CA, USA, 1 : 100) followed by incubation with HRP-conjugated secondary antibodies (Abcam, MA, USA, 1 : 5,000) for 2 h at room temperature. Signals were visualized with an enhanced chemiluminescence detection kit (Beyotime, Shanghai, China) and results were analyzed with National Institutes of Health Image 1.41 software.

### 2.15. Statistical Analysis

All data were expressed as mean ± SD. Data were analyzed by one-way ANOVA with Tukey's post hoc test or by two-way ANOVA with Bonferroni post hoc test. Statistical analysis was performed using the SPSS 16.0 statistics software (SPSS, Chicago, IL), and *P* < 0.05 was considered statistically significant.

## 3. Results

### 3.1. PTEN Is Highly Expressed in the Ipsilateral Hippocampus after ICH

**Figure 1:**
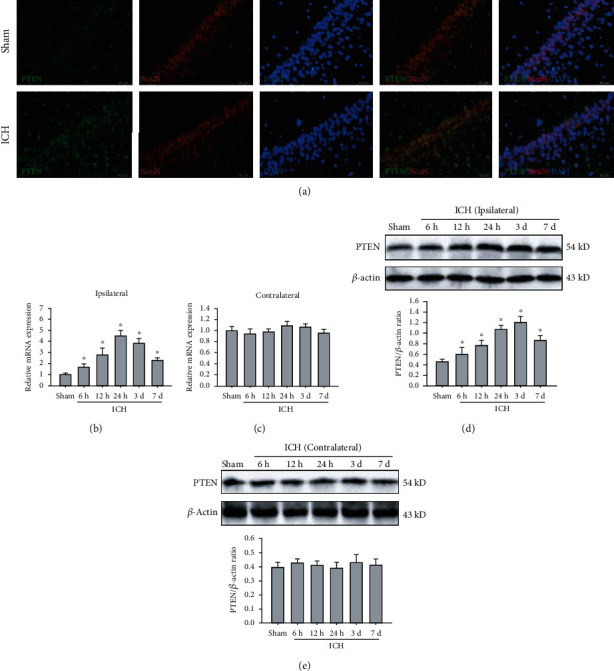
ICH caused an increase in endogenous PTEN in the ipsilateral hippocampus. (a) Representative images of double immunofluorescence staining for PTEN (green) and NeuN (red) in the hippocampal CA1 region. Bar, 50 *μ*m. (b, c) The mRNA level of PTEN in the ipsilateral (b) and contralateral hippocampus (b) were examined by real-time PCR. GAPDH serves as a loading control. (d, e) The protein expression of PTEN in the ipsilateral (d) and contralateral hippocampus (e) was measured by Western blot. *β*-Actin was used as a loading control. ^∗^*P* < 0.05 compared with the Sham group.

It has been proposed that PTEN preferentially expresses in neurons in the brains of adult mice, especially large pyramidal neurons [19]. First, we detected the cellular localization of PTEN in hippocampal CA1 regions of rats. Double immunofluorescence staining showed that PTEN protein was exclusively localized in the cytoplasm of hippocampal pyramidal neurons, but rarely expressed in astrocytes (Figure 1(a)). To further assess the expression pattern of PTEN in the ipsilateral and contralateral hippocampus after basal ganglia hemorrhage, we identified that the mRNA and protein levels of PTEN were significantly upregulated in the ipsilateral hippocampus, but not altered in the contralateral hippocampus after ICH (Figures 1(b)–1(e)). Specifically, the PTEN expression was markedly increased at 6 h, peaked at 24 h in the transcription level (3 d in the translation level), and then gradually decreased after ICH. Collectively, ICH can induce a striking upregulation of PTEN in the ipsilateral hippocampus in rats.

### 3.2. PTEN Inhibition Attenuates Poststroke Cognitive Impairments

**Figure 2:**
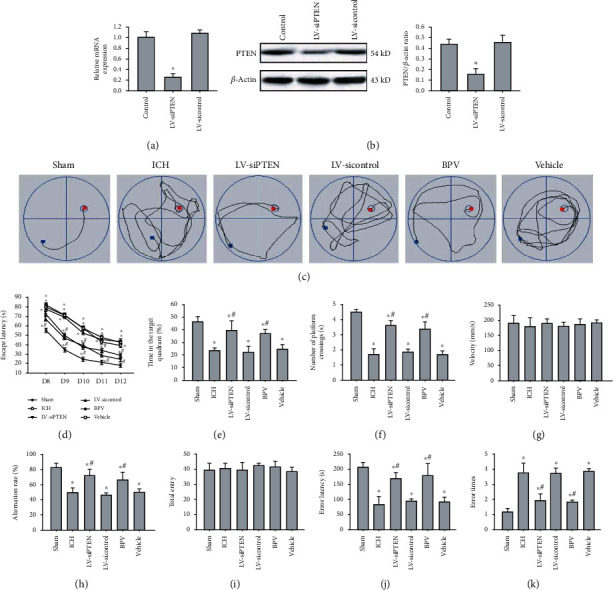
PTEN deletion improved ICH-induced cognitive deficits. (a, b) Silencing efficiency of PTEN was detected by real-time PCR and Western blot analysis at 72 h after infection. (c) Representative images showed swim paths on days 12 after ICH. (d–g) Escape latency (d), target quadrant time (e), times across the platform (f), and velocity (g) were analyzed in the MWM test at 8-13 d postinjury. (h, i) Spatial working memory was assessed using the Y-maze test by monitoring spontaneous alteration at 7 d postinjury. (j, k) Latency to reenter the dark compartment (error latency) and error times were evaluated in the PAT test at 14 d post-ICH. The data are represented as the mean ± SD of five independent experiments. ^∗^*P* < 0.05 compared with the Sham group; ^#^*P* < 0.05 compared with the ICH group.

To evaluate the functional roles of elevated PTEN in the experimental ICH model, we employed PTEN molecular inhibitor BPV and PTEN siRNA to suppress endogenous PTEN expression in rats. As expected, hippocampal injection of LV-siPTEN strongly downregulated the mRNA and protein level of PTEN in hippocampal tissues relative to the control group and the LV-sicontrol group (Figures 2(a) and 2(b)). We next investigated the impacts of PTEN deletion in ICH-mediated cognitive impairments using a series of behavioral tests. Compared with the Sham group, ICH rats displayed a striking spatial learning and memory loss, as evidenced by significant longer escape latencies (Figures 2(c) and 2(d)), decreased time spent in the target quadrant (Figure 2(e)), and the number of platform crossings (Figure 2(f)) in the MWM test. These effects, however, were obviously prevented by systemic administration of dipotassium bisperoxo (picolinato) oxovanadate (BPV) or hippocampal injection of LV-siPTEN. Given subtle residual motor dysfunction might influence animal performance, we also recorded swimming velocity and found no significant difference between the groups (Figure 2(g)). Subsequently, the Y-maze test was conducted to monitor spatial working memory in rats at 7 d post-ICH. We noted the spontaneous alternative ratio of ICH rats was lower than that in the Sham group, whereas blockage of PTEN could significantly improve spontaneous alternations after ICH (Figure 2(h)). However, there was no significant difference in the total number of arm entries between groups (Figure 2(i)). Furthermore, in the PAT task, we observed that rats treated with BPV or LV-siPTEN have longer latency and less error times to enter the dark compartment than that in the ICH group, indicating the improvement of their retention of nonspatial memory (Figures 2(j) and 2(k)). Taken together, PTEN inhibition may contribute to hippocampal-dependent learning and memory improvement.

### 3.3. PTEN Inhibition Reverses Secondary Hippocampal Injury Post-ICH

**Figure 3:**
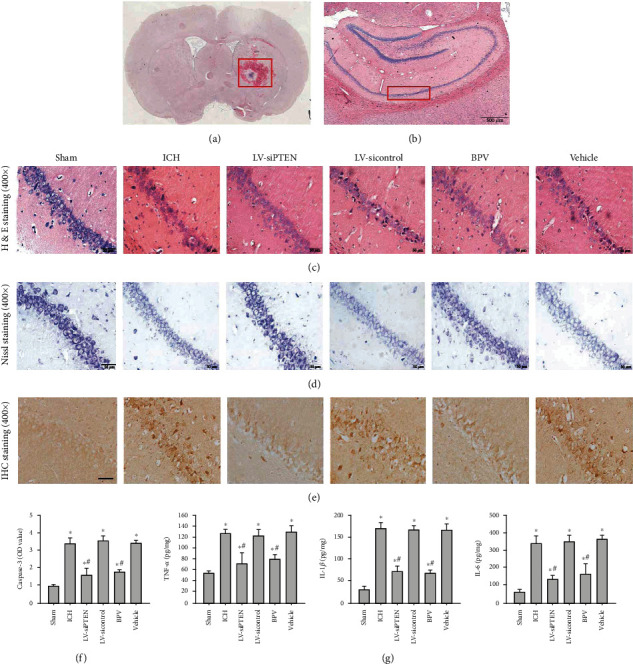
PTEN knockdown attenuated ICH-induced secondary hippocampal injury. (a–e) Representative images of H&E staining (a, b, c), Nissl staining (d), and caspase-3 IHC staining (e) in the hippocampal CA1 region at 3 d after ICH in rats. Images in (a) and (b) showed the site of brain hemorrhage and the CA1 region of the hippocampus, respectively. Bar, 50 *μ*m. (f) The quantitative analysis of caspase-3 protein normalized to the control group. (g) The impacts of PTEN inhibition on hippocampal neuroinflammation by analyzing the contents of TNF-*α*, IL-1*β*, and IL-6 in the ipsilateral hippocampus using ELISA analysis at 3 d postinjury. ^∗^*P* < 0.05 compared with the Sham group; ^#^*P* < 0.05 compared with the ICH group.

Next, we elucidated the effects of PTEN knockdown on poststroke hippocampal injury caused by basal ganglia hemorrhage in rats. The morphological analysis illustrated that hematoma triggered secondary injury to the ipsilateral hippocampus manifested by structural abnormalities and neuronal damage of the hippocampal CA1 region at 3 d post-ICH (Figures 3(a)–3(d)), whereas no significant morphologic changes were noted in the contralateral hippocampus. Importantly, pharmacological and genetic inhibition of PTEN prominently reduced secondary hippocampal injury in rats subjected to ICH. Furthermore, we measured neuronal apoptosis and neuroinflammatory response in the ipsilateral hippocampus by immunohistochemistry and ELISA analysis at 3 d following ICH. The results indicated that the expression of apoptosis-associated signal protein caspase-3 (Figures 3(e) and 3(f)) and the contents of inflammatory cytokines including tumor necrosis factor- (TNF-) *α*, interleukin- (IL-) 1*β*, and IL-6 (Figure 3(g)) were remarkably reduced by BPV treatment or LV-siPTEN infection. Overall, these data strongly suggest that endogenous PTEN knockdown protects against secondary hippocampal damage after experimental ICH.

### 3.4. PTEN Inhibition Blocks ICH-Induced Excessive Activation of Neuronal Autophagy

**Figure 4:**
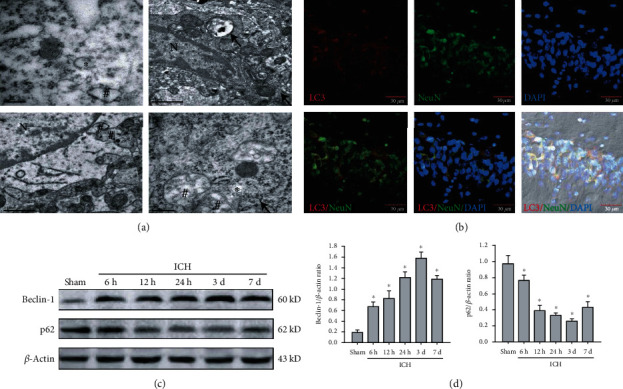
ICH triggered autophagy activation in the nonhemorrhagic hippocampus. (a) Representative images of electron microscopy in the ipsilateral hippocampus after ICH. The asterisk symbol indicated phagophore, the hash symbol presented typical autophagosomes, and the arrow showed the formation of autolysosomes. Bar, 1 *μ*m. (b) The colocalization of LC3 (red) in the neuron (green) of the hippocampal CA1 region after ICH. Bar, 30 *μ*m. (c, d) Representative bands and quantitative analysis of Beclin-1 and p62 in the ipsilateral hippocampus. ^∗^*P* < 0.05 compared with the Sham group; ^#^*P* < 0.05 compared with the ICH group.

**Figure 5:**
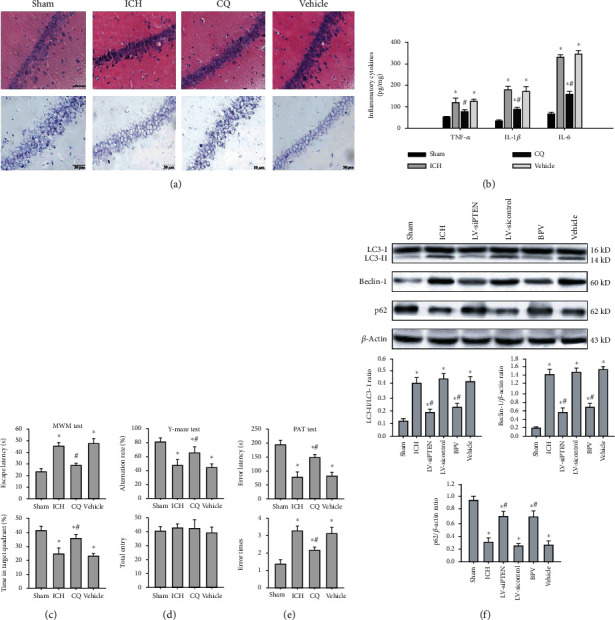
PTEN knockdown prevented hemorrhagic outcomes via suppressing neuronal autophagy. (a) Representative images of H&E and Nissl staining in the hippocampal CA1 region at 3 d post-ICH. Bar, 50 *μ*m. (b) ELISA analysis of inflammatory cytokines TNF-*α*, IL-1*β*, and IL-6 in the damaged hippocampus. (c–e) The effects of CQ on hippocampus-dependent learning and memory were assessed in the MWM test, Y-maze test, and PAT test. (f) Representative bands and quantitative analysis of LC3, Beclin-1, and p62 in the ipsilateral hippocampus post-ICH. ^∗^*P* < 0.05 compared with the Sham group; ^#^*P* < 0.05 compared with the ICH group.

To clarify the potential mechanisms responsible for this important neuroprotection of PTEN knockdown, we investigated the alterations and roles of the autophagic program in rat models of ICH. Images from electron microscopy, considered the most accurate method to monitor autophagy, clearly exhibited the presence of phagophore autophagosomes and autolysosomes together with extensive cytoplasmic vacuolization in the CA1 region of ipsilateral hippocampus postinjury (Figure 4(a)). Microtubule-associated protein light chain-3 (LC3) is well recognized as an important autophagic biomarker. During autophagy, cytosolic LC3-I converted to their lapidated form LC3-II that incorporated in the autophagosome. Immunofluorescent results demonstrated that a large number of LC3 puncta was observed in the cytoplasm of hippocampal CA1 neurons (Figure 4(b)). Next, we detected the expression of autophagy-related proteins, including Beclin-1 and p62 in hippocampal tissues. Following ICH, we found that the Beciln-1 expression was markedly increased, whereas the p62 level was decreased in a time-dependent manner in hippocampal tissues (Figure 4(c)). The above data confirmed that ICH might strongly initiate neuronal autophagy, but the function of autophagy still remains unclear. Notably, administration of CQ, an autophagy inhibitor, obviously relieved posthemorrhagic hippocampal lesion and neuronal damage (Figure 5(a)), inflammatory response (Figure 5(b)), and delay cognitive decline (Figures 5(c)–5(e)), suggesting a harmful autophagy form of programmed cell death after ICH. More importantly, blockage of PTEN could remarkably inhibit ICH-induced autophagy manifested by decreased LC3-II to LC3-I ratio and Beclin-1 protein and increased p62 level in the ipsilateral hippocampus (Figure 5(f)). Therefore, we conclude that PTEN knockdown conferred potent neuroprotective effects against hemorrhagic outcomes through suppressing excessive activation of neuronal autophagy.

### 3.5. PTEN Inhibition Increases FoxO3a Phosphorylation and Blocks Its Nuclear Translocation Involving the PI3K/AKT Pathway

**Figure 6:**
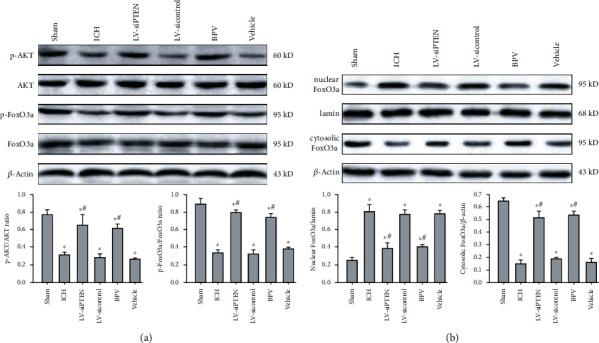
PTEN inhibition restricted FoxO3a dephosphorylation and nuclear translocation by the PI3K/AKT signaling. (a, b) Representative bands and quantitative analysis of p-AKT, AKT, p-FoxO3a FoxO3a, and nuclear/cytosolic FoxO3a in the ipsilateral hippocampus at 3 d post-ICH. Lamin was used as a nuclear fraction loading control. ^∗^*P* < 0.05 compared with the Sham group; ^#^*P* < 0.05 compared with the ICH group.

We sought to decipher the molecular mechanisms underlying PTEN knockdown-mediated autophagy inactivation. Forkhead box O (FoxO) 3a, a critical transcription factor, is also considered as an autophagy-regulatory protein and can be phosphorylated and inactivated by the PI3K/AKT cascade. Poststroke elevated PTEN, a nature antagonist of the PI3K/AKT signaling, was accompanied by a decrease in the phosphorylation and activity of AKT in the ipsilateral hippocampus of rats. Subsequently, inactive AKT reduced its downstream substrate FoxO3a phosphorylation modification, leading to the enhancement of FoxO3a nuclear translocation (Figure 6(a)). Further study demonstrated that cytosolic FoxO3a level in the ICH group was markedly decreased, but nuclear FoxO3a was apparently increased in comparison with the Sham group (Figure 6(b)). In contrast, BPV treatment or PTEN silencing resulted in a significant elevation of p-AKT and p-FoxO3a level, leading to FoxO3a cytoplasmic accumulation and subsequent degradation. However, there was no significant difference in FoxO3a and AKT protein between the groups. To sum up, these evidences unveil that the PI3K/AKT/FoxO3a axis plays an important role in PTEN deletion-mediated autophagic inactivation after ICH.

### 3.6. Alterations of FoxO3a Are Essential for the Antiautophagic Effects of PTEN Inhibition

**Figure 7:**
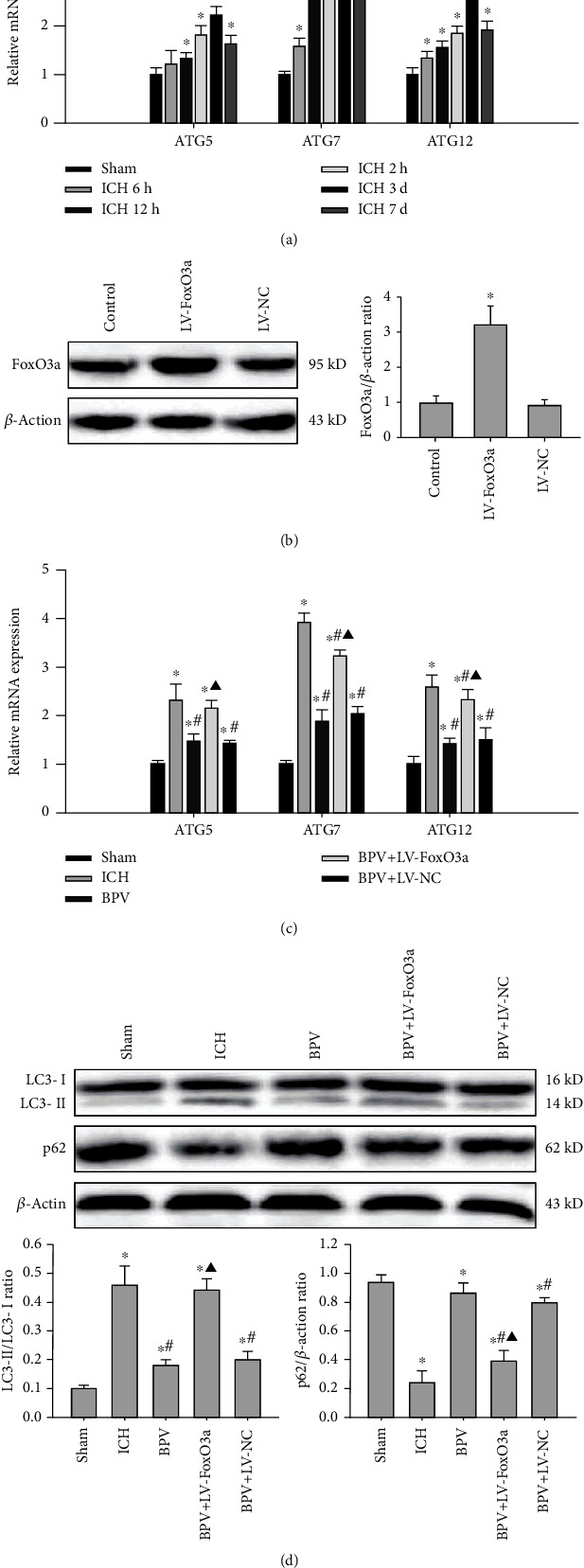
FoxO3a was required for the negative regulatory effects of PTEN on the autophagic program. (a) The mRNA of ATG5, ATG7, and ATG12 in the ipsilateral hippocampus was detected by real-time PCR. (b) Overexpression efficiency of FoxO3a was detected by Western blot at 72 h after infection. (c, d) The effects of FoxO3a overexpression on PTEN deletion-mediated autophagy inactivation were assessed by ATG mRNA levels as well as LC3 and p62 protein expression. ^∗^*P* < 0.05 compared with the Sham group; ^#^*P* < 0.05 compared with the ICH group; ^▲^*P* < 0.05 compared with the BPV group.

To examine whether FoxO3a is required for PTEN-mediated negative regulation of autophagy, we analyzed the mRNA levels of ATG5, ATG7, and ATG12 in the ipsilateral hippocampus post-ICH. Real-time PCR results indicated that ATGs were dramatically elevated at hour 6, peaked at day 3, and gradually decreased at day 7 postinjury (Figure 7(a)). This time-dependent upregulation of ATG genes resulted from the enhancement of FoxO3a nuclear translation and transcription activity. After PTEN inhibition, the mRNA levels of ATG5, ATG7, and ATG12 were remarkably reduced in the ipsilateral hippocampus. According to these data, we speculate that posthemorrhagic PTEN elevation triggers the nuclear accumulation of FoxO3a and subsequent transcriptional activation of ATGs, resulting in sequential activation of autophagy. More importantly, lentivirus-mediated FoxO3a overexpression effectively abrogated the antiautophagic effects of PTEN inhibition, as evidenced by elevated ATG mRNA levels, increased the ratio of LC3-II to LC3-I, and decreased the p62 expression after ICH (Figures 7(b)–7(d)). Overall, FoxO3a was an indispensable effector for the inhibitory effect of PTEN deletion on the autophagic program after a hemorrhagic stroke.

## 4. Discussion

Understanding potential mechanisms of poststroke distal injury and cognitive impairment is a fundamental question, with important implications for developing effective therapeutic targets. This study demonstrates for the first time that (1) pharmacological and genetic inhibition of PTEN alleviated secondary hippocampal injury and delayed cognitive deficits in rat models of basal ganglia hemorrhage. (2) These potent neuroprotective effects of PTEN knockdown were achieved by suppressing excessive activation of autophagy in the ipsilateral hippocampus. (3) PTEN-mediated proautophagic program was dependent on the PI3K/AKT/FOXO3a/ATG axis following ICH.

Beyond the primary mechanical injury and motor dysfunction, ICH also leads to secondary injury to distal brain regions and delayed cognitive deficits. Although the hematoma commonly occurs in the basal ganglia, the hippocampus, an extremely sensitive and vulnerable region, often suffers secondary injury. Moreover, secondary hippocampal injury is recognized as a main cause of impaired cognition and memory. Here, we established the ICH model by injection of autologous whole blood into the right basal ganglia of adult rats. Following ICH, we observed ipsilateral hippocampal damage together with a subsequent decline of cognitive function. Although many studies have focused on neurological outcomes after a hemorrhagic stroke, but precise mechanisms responsible for secondary hippocampal and cognitive loss are not fully elucidated. Recent studies document that aberrant maturation of newly generated neurons and an increase in sharp-wave-associated ripples in the hippocampus contribute to defective cognition after ischemic stroke [28, 29]. Other evidence also shows that impaired adult hippocampal neurogenesis is detrimental to the spatial learning and memory after basal ganglia hemorrhage [8]. Furthermore, a striking cognitive impairment is accompanied by reduced synaptic plasticity and dendrite spine density of ipsilateral hippocampal neurons [10]. Intriguingly, our current study revealed that secondary hippocampal disturbances were accompanied by robust activation of neuronal autophagy after ICH. Suppression of autophagy by CQ could efficiently relieve functional and histological outcomes of the ipsilateral hippocampus. Hence, ICH-mediated autophagic cell death in hippocampal neurons shaped long-term cognitive decline, but the upstream events that initiate autophagy remain unclear.

ext, PTEN, a positive autophagy-regulatory factor, was found to exclusively localize in the cytoplasm of the hippocampal CA1 pyramidal neuron and highly express in the ipsilateral hippocampus after ICH. An important significance of PTEN has been demonstrated in local and global modulation of neuronal function in health and disease [30]. Abnormal expression and activity of PTEN gives rise to synaptic dysfunction with behavioral and cognitive consequences in Alzheimer's models [31]. Functionally, our findings confirmed that inhibition of PTEN by PTEN siRNA or specific inhibitor not only ameliorated secondary hippocampal injury but also promoted hippocampal-dependent cognition and memory recovery, suggesting important neuroprotective effects against hemorrhagic insults. More importantly, we validated the potent neuroprotection of PTEN knockdown was achieved by inhibiting ICH-induced autophagic cell death in the ipsilateral hippocampus of rats. To date, the underlying mechanisms of PTEN-mediated excessive autophagic program post-ICH remain obscure. Notably, in A549 and HeLa cells, phosphorylation of PTEN at serine 113 contributed to PTEN nuclear accumulation and the subsequent autophagy initiation [32]. In contrast, posthemorrhagic upregulation of PTEN protein mostly resided in the cytoplasm of hippocampal neurons. Consequently, we speculate that the potent proautophagic effect of PTEN may be the involvement of distinct molecular mechanisms following ICH.

In the present study, PTEN inhibition not only reduced the levels of autophagy-related proteins but also activated the PI3K/AKT pathway in the ipsilateral hippocampus after ICH. Specifically, PTEN dephosphorylates phosphatidylinositol 3, 4, 5-trisphosphate (PIP3) to PIP2, thereby antagonizing the activity of PI3K/AKT signaling [19]. It has been well recognized the PI3K/AKT signaling and its downstream substrate mTOR are major negative regulators of autophagy [33]. Nevertheless, FoxO3a, a member of the FoxO family of transcription factors, is an additional downstream effector. The transcriptional activity of FoxO3a is regulated by phosphorylation and subcellular localization. Phosphorylated FoxO3a remains in the cytoplasm, and unphosphorylated FoxO3a translocates to the nucleus and initiates transcription of downstream genes via a transactivation/chromatin remodeling domain (TAD) [34]. FoxO3a is phosphorylated by active AKT and retained in the cytosol, thereby inhibiting its transcriptional activity. Our current data uncovered that ICH resulted in an increase in nuclear FoxO3a protein and a decrease in cytosolic FoxO3a and phosphorylated FoxO3a, but hardly affected total protein level in hippocampal tissues. Indeed, ICH-mediated upregulation of PTEN repressed the AKT activity and then facilitated FoxO3a dephosphorylation and nuclear translocation. Interestingly, all posthemorrhagic alterations of FoxO3a were diminished by endogenous PTEN inhibition. These data revealed that PTEN only regulated FoxO3a phosphorylation, activity, and subcellular localization and rarely impacted its total protein expression.

Intriguingly, it has been recently proposed that FoxO3a coordinately activates autophagy by binding directly to the promoters of ATGs or autophagy regulatory genes [35]. Mutation of FoxO3a phosphorylation sites accelerates its nucleus translocation and subsequent autophagy initiation, and FoxO3a knockdown also may induce a striking reduction of the proautophagic program [36]. Herein, we identified that ICH induced a significant increase in ATG transcriptional levels including ATG5, ATG7, and ATG12, which was strongly associated with PTEN-mediated FoxO3a nuclear translocation. However, blockage of PTEN prominently abolished these ATG transcriptions and subsequent autophagy induction. Importantly, we found that the overexpression of FoxO3a obviously abrogated the suppression of the proautophagic program mediated by PTEN inhibition after ICH. These data strongly suggested that FoxO3a was an essential effector for potential antiautophagic effects of PTEN deletion. More interestingly, it has been shown that FoxO3 is itself a substrate for basal autophagic degradation, and autophagy may regulate FoxO3a turnover and transcriptional activity [37, 38], which adds another interesting feature to the mechanisms.

In conclusion, these findings demonstrated that PTEN inhibition effectively relieved ICH-induced secondary hippocampal injury via suppressing neuronal autophagy, eventually improving delayed cognitive deficits. Mechanistically, blockage of PTEN could enhance FoxO3a phosphorylation modification to restrict its nuclear translocation and ATG transcription via activating the PI3K/AKT pathway, leading to the suppression of the autophagic program. Therefore, this study has an important implication for the development of effective therapeutic targets for ICH.

## Data Availability

The data used to support the findings of this study are included within the article.
